# Implementation strategies to improve adoption of unmet social needs screening and referrals in care management using enabling technologies: study protocol for a cluster randomized trial

**DOI:** 10.21203/rs.3.rs-4985627/v1

**Published:** 2024-10-17

**Authors:** Nicole Cook, Rose Gunn, Brenda M. McGrath, Jenna Donovan, Maura Pisciotta, Constance Owens-Jasey, Hannah L. Fein, Anna Templeton, Zoe Larson, Rachel Gold

**Affiliations:** Ochin; Ochin; Ochin; Ochin; Ochin; Ochin; Ochin; Ochin; Ochin; Ochin

**Keywords:** care management, clinical-community linkages, social determinants of health, health disparities, primary care, safety-net populations

## Abstract

**Background:**

Adverse social determinants of health contribute to health inequities. Practice guidelines now recommend incorporating patient unmet social needs into patient care, and payors increasingly reimburse for screening and providing related referrals to community organizations. Emergent electronic health record (EHR)-based tools can enable clinical-community linkages, but their adoption commonly faces workflow and infrastructure barriers. Targeted implementation support such as training, championship, practice facilitation, and audit and feedback, can enhance such tools’ adoption, but no prior research has assessed such strategies’ impact on the adoption of ‘enabling technologies’ supporting clinical-community linkages. This study will test whether providing targeted implementation support to safety-net primary care health center care management teams improves the sustained adoption of EHR-based enabling technologies used to 1) screen for social needs and 2) link patients to community organizations.

**Methods:**

Formative evaluation of barriers and facilitators to adopting EHR-enabled social needs referrals and ascertainment of services received will include semi-structured interviews and a ‘guided tour’ of enabling technology used by care managers serving patients with complex health and/or social needs. A modified Delphi process conducted with care management staff and subject matter experts will then inform the development of an intervention targeting adoption of social risk EHR-enabled tools. The intervention will be piloted in three health centers, refined, then tested in a pragmatic stepped-wedge cluster-randomized trial in 20 health centers (five wedges of four health centers) that provide care management to high-risk patients with social needs.

**Discussion:**

This study is among the first to evaluate an intervention designed to support care management teams’ adoption of enabling technologies to increase clinical-community linkages. It was funded in September 2023 by the National Institute of Nursing Research. Formative activities will take place from January to June 2024, the intervention will be developed in July-December 2024, the pilot study will be conducted from January-March 2025, and the cluster-randomized trial will occur from July 2025 -September 2026. Study data will be analyzed and results disseminated in 2027–2028. Study results have the potential to improve clinical-community linkages and in so doing to advance health equity.

**Trial registration:**

Clinicaltrials.gov registration # NCT06489002. Registered July 5, 2024, https//clinicaltrials.gov/study/NCT06489002?term=NCT06489002&rank=1

## Background

Decades of research show that adverse social determinants of health are disproportionately prevalent in the low-income, racially / ethnically / geographically diverse populations served by the nation’s health care safety net. Patients’ unmet social needs, including food, housing, financial, and transportation insecurity, contribute to sociocultural and contextual barriers to chronic disease management(1–3) and thus to disparities in health outcomes.(3–5)

A growing body of evidence supports the potential benefits of healthcare providers referring patients to community organizations to address their unmet social needs,(6–9) and numerous experts recommend addressing social needs in this way as part of comprehensive care for prevalent chronic diseases like hypertension and diabetes.(10–12) Adoption of such ‘social needs-targeted care’ activities is further encouraged through emergent value-based payment models that require unmet social needs screening and the provision of referrals to community organizations, and other national health equity initiatives that emphasize addressing social needs as part of care delivery.(13–18) Care management and population health programs (referred to hereafter as “CM”), which support whole-person, patient-centered coordinated care for high-risk and medically complex patients (e.g., Medicare’s Chronic Care Management program and the California Advancing and Innovating Medi-Cal (3–4) [CalAIM] programs) also emphasize care coordination via referrals to community organizations to address patients’ health-related social needs.(9, 19, 20)

Such clinical-community linkages are increasingly supported by ‘enabling technologies’ such as electronic health record (EHR)-based care coordination applications and care management software products. For example, Compass Rose (CR) is an EHR-integrated application designed to support care coordination and care management activities, including assessing patients’ social needs, referring patients to community organizations, and tracking referral outcomes.(21, 22) While these new technologies have great promise at supporting clinical-community organizational linkages, little is known about effective strategies for optimizing their systematic and sustained adoption, especially in safety-net clinical care settings serving complex patients.(8, 23) Developing effective strategies for supporting clinical-community linkages requires understanding the barriers to using enabling technologies in these care settings and testing strategies designed by health center and community partners to address these barriers. One known barrier is the up-front investment in workflow redesign and technology infrastructure needed to use these technologies to facilitate bi-directional referrals.(24–28) Few care management and population health reimbursement mechanisms cover these investments, which is a barrier to under-resourced primary care teams’ ability to adopt enabling technologies that could support clinical-community linkages.

Evidence from the implementation of other care management programs(29–33) and health information technologies (HIT)(34, 35) may inform operationalizing the adoption of such enabling technologies. Evidence-based implementation strategies shown to advance the adoption of other technologies in health centers include leadership support,(36) staff training,(37–40) local champions,(41–45) practice facilitation,(46) and audit and feedback.(29–33) Applying these strategies effectively requires consideration of the specific needs and context of different health centers as well as barriers specific to the adoption of enabling technologies to support clinical-community linkages.(29–32, 47, 48) It is critical to engage health center users as partners to guide the development of a set of evidence-based implementation strategies to address such barriers. A structured approach to interactively fine-tuning implementation strategies is needed to develop an implementation support intervention that can then be formally tested to generate evidence for replication across health centers.

This paper describes the protocol for a National Institutes of Health (NIH)-funded study called DEDICATE: Advancing Care Management Adoption in Community Health Centers (R01NR021115). The study will develop and evaluate the effectiveness of implementation strategies designed to support care management teams’ sustained use of EHR-based functionalities that support clinical-community linkages. It will test the hypothesis that health center partners’-adapted implementation strategies applied to enabling technologies will improve the adoption of these technologies and thus social risk screening, community referrals, and receipt of services by patients with unmet social needs. The study’s overarching goal is to improve clinical-community linkages through care management and care coordination programs that serve high-risk patients with complex health and/or social needs.

## Methods

### Setting

This study will be conducted among health centers that are members of OCHIN, Inc., a multi-state network of community health organizations that serve more than 6 million people in rural and medically underserved communities nationwide. OCHIN is a national nonprofit health IT consultancy that provides a fully hosted and shared EHR platform with tailored tools and expertise, including practice management tools, to > 1,000 care delivery sites across 30 states. While all OCHIN members share a single instance of the Epic EHR, each is an independent organization with its own governing board. Conducting this study in a diverse group of health centers improves the likelihood that its results will be generalizable to other primary care settings, an important feature of pragmatic trials.

OCHIN Epic includes a robust suite of tools designed to support collecting and acting on social risk data, many developed through NIH-funded studies.(28, 49–54) One such tool, CR, was made available to OCHIN members in 2022. CR is an integrated EHR tool that supports comprehensive care coordination for high-touch care management, including assessing patients’ social needs, making referrals to community organizations, and tracking referral outcomes to ascertain receipt of community services. Yet at present, an estimated < 10% of health centers use these functionalities to make referrals and track referral outcomes.

All OCHIN health centers that have activated the CR tools in their EHR and participate in one or more CM, population health, or value-based payment model programs that require care coordination of social needs for patients will be eligible for the trial. Such programs include CMS’s care management programs, CalAIM, and patient-centered medical home (PCMH) programs. At least 42 OCHIN member health centers met these criteria at study start-up (November 2023) and another six had adopted CR tools by March 2024. We anticipate that even more health centers do so and thus be eligible for recruitment in early 2025.

### Conceptual Model

The study will test a suite of implementation support strategies (training, champion, practice facilitation, and audit and feedback). They will be refined in the study’s formative phase, per guidance from health center partners and technology subject matter experts, to optimize how they support the provision of clinical-community linkages by CM teams. Intervention development and study outcomes are guided by the Integrated Technology Implementation Model (ITIM)(55, 56) which considers facilitators and barriers to technology adoption and applies quality improvement methods to facilitate and sustain adoption. Its intended use is to support a deep understanding of the interplay of internal and external concepts important to implementing enabling technologies (such as the CR application). This knowledge will support the selection and iterative refinement of the implementation strategies comprising the study intervention. Study concepts mapped to ITIM concepts are outlined in Table 1.

Study outcomes are guided by RE-AIM (Reach, Effectiveness (impact), Adoption, Implementation and Maintenance) and RE-AIM QuEST (Qualitative Evaluation for Systematic Translation). Both are widely used frameworks for informing and assessing key dimensions of intervention development and measuring intervention implementation.(57–59)

### Formative Intervention refinement

The planned key components of the intervention will include 12 weeks of evidence-based implementation support strategies expected to include training, championship, practice facilitation, and audit and feedback; these were selected based on evidence that they support the adoption of similar HIT tools.

**Figure 1:**
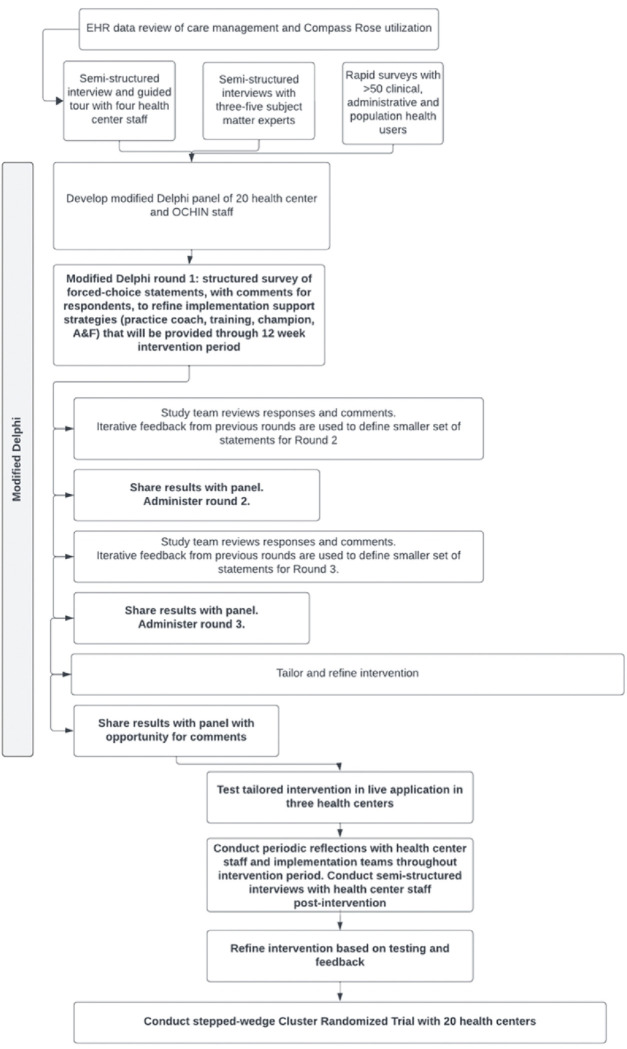
DEDICATE formative activities

A formative mixed methods approach will focus on understanding how individual, health center, and environmental factors influence care team use of enabling technologies to support social care coordination activities, and how the implementation strategies to be tested can optimally promote such technologies’ adoption. The implementation strategies and expected refinements to be informed through formative ethnographic methods is in Table 2. Formative activities that will occur during the first six months of the study are shown in Fig. 1 and described below.

To ensure a comprehensive understanding of CR utilization for social care coordination activities, we will first review OCHIN network data. These data will be extracted from the OCHIN Epic EHR and used to quantify the number of patients at each OCHIN clinic who were enrolled in specific CR CM programs including Transitional Care Management (TCM), Chronic Care Management (CCM), Principal Care Management (PCM), California Advancing and Innovating Medi-Cal (CalAIM), and Patient-Centered Medical Home (PCMH). Clinic patient volume, clinic social risk screening rates, and the number of CR patients with social risk screens will be assessed. Finally, procedure codes associated with billing for the above-mentioned Centers for Medicare and Medicaid (CMS) care management programs will be extracted as a potential way of assessing care management occurring in these health centers.

The study team will review these data with quality improvement practice coaches who support social needs-targeted care at OCHIN. We will collaboratively identify and recruit four diverse health centers to explore their use of CR for social care coordination activities via ethnographic qualitative methods.(60–63) Eligible health centers include those with at least 20 patients per month enrolled in CR programs during September 1, 2023 to November 30, 2023. Recruitment will be based on 1) number of patients enrolled in CR programs, 2) participation in care models that incorporate social needs care coordination (e.g. CalAIM, CCM), 3) percentage of patients with social risk screening, and 4) geographic diversity.

To recruit diverse health centers, we will create three strata. The first stratum will comprise health centers that use CR programs other than CalAIM and have a high social risk screening rate (rates in the upper 50th percentile of all health centers with ≥ 20 CR patients). The second stratum will include health centers that use CR programs other than CalAIM and have a lower social risk screening rate (screening rates in the lower 50th percentile among all health centers with 20 or more CR patients). The third stratum will include any other health centers that use CR programs including CalAIM.

The first stratum includes eight eligible health centers, among them four from states other than CA. We will prioritize the health centers outside of CA for this stratum to maximize geographic diversity but will recruit a CA health center if needed. The second stratum includes 18 eligible health centers, and we aim to recruit two of these. We will prioritize centers within the second stratum based on geographic distribution and feedback from the study team. In the third stratum of nine health centers utilizing CR for CalAIm alongside other programs, two were prioritized based on their use of CalAIm plus another program and study team input regarding past and concurrent collaborations. The remaining health centers in this stratum were reserved for potential recruitment in subsequent rounds, if needed.

Representatives of the four recruited health centers will participate in a one-hour semi-structured interview. Informed by prevalent human factors engineering and human-centered design principles, interviews will include a “guided tour” of CR and other related EHR tools to understand how enabling technologies are currently used to support social needs activities. Interviews will explore implementation barriers and facilitators related to the reach, effectiveness, and adoption of such enabling technologies for social risk screening and clinical-community linkages. Participants identified by the recruited health centers are likely to include care managers, community health workers, and persons in similar roles.

Concurrently, the study team will conduct semi-structured interviews with five OCHIN subject matter expert staff, including practice coaches, population health leaders, and clinical informatics experts who work with health centers to support the adoption of workflows and technologies to facilitate social needs-targeted care.

The study team will also seek feedback via rapid, online surveys (< 5 questions) from hundreds of OCHIN member health center clinical, administrative, and population health users who attend standing workgroup and committee meetings. Results from these rapid surveys will further the study team’s understanding of facilitators and barriers of clinics’ adoption of the EHR tools of interest.

Quantitative and ethnographic qualitative data collected during formative activities will be integrated and examined using a convergent triangulation design, guided by the ITIM and RE-AIM QuEST frameworks, to relevant explicate patterns, barriers, and facilitators. Qualitative data analysis will use a framework-driven rapid analytic approach to produce rich findings to identify contextual barriers and facilitators related to the adoption of CR and other EHR tools for social risk screening and referrals and inform refinement of the intervention. This approach supports the rapid identification of key insights into the applicability and feasibility of implementation strategies that will be further iterated through a modified Delphi process.(31, 64)

The modified Delphi process conducted subsequently will obtain community partner consensus on the operationalization of related intervention strategies in study health centers. Process participants will include health center staff from the four health centers participating in the formative phase described above, and additional health center staff that express interest following rapid presentations at relevant OCHIN member workgroups and committees. Participants will also include OCHIN subject matter experts in practice coaching, clinical informatics, population health, and learning content development.

The modified Delphi process will include three sequential rounds of virtual surveys administered via REDCap with statements on the potential implementation strategies’ content, format for delivery, and cadence, with multiple-choice responses and a free text option. For example: “Training on optimizing use of enabling technologies to coordinate care for social needs among care management patients should include (choose as many as apply): a) real-time support; b) weekly demos; c) online modules; d) drop-in office hours.” Participants will also be able to suggest implementation strategies other than those presented.

After each survey round, members of the research team will analyze results, remove or revise statements that did not receive at least 70% agreement, and modify other statements based on participant comments to iteratively build consensus on how implementation strategies will be applied. Summary data of each round will be given in an easily interpretable format to all modified Delphi participants before the next round. Following round three, the proposed implementation strategies will be shared with participants with an opportunity for comment, after which the study team will finalize the first intervention iteration.

Next, we will further refine the intervention by pilot-testing it in three health centers. We will recruit health centers that, in the prior month, 1) used any CR functions for ≥ 15 patients; 2) participate in at least one care management or care coordination program that requires social risk screening and referrals, and 3) express interest in providing feedback on application of CR to support clinical-community organization linkages. We will conduct a baseline survey with health center care management / population health leadership to assess readiness, context, barriers, and external factors related to care coordination using enabling technology. The survey’s open and closed-ended questions will be informed by the ITIM framework and administered via REDCap.

The intervention will involve delivering the implementation strategies over 12 weeks to health center care teams. During this period the study team will collect qualitative data through periodic reflections with the health centers’ implementation teams during regularly scheduled check-ins. Periodic reflections are guided discussions informed by ethnographic methods and will be conducted twice during the intervention period at weeks 4 and 8. We anticipate that each health center will implement site-specific adaptations to the intervention to increase its effectiveness within the local context. Data collected will provide detailed, systematically captured information on intervention activities, implementation settings, contextual factors, adaptations to the proposed intervention, and implementation team sense-making and learning. Post-intervention semi-structured interviews with two to three clinic staff per health center will also be conducted to understand experiences with intervention implementation, recommendations for future implementations, and potential implementation strategy support adaptions that should be considered.

These formative activities will support a deep understanding of barriers to the adoption of enabling technologies by engaging with health center partners to identify which support strategies are likely to address such barriers and the specifics of how they should be implemented (e.g., the cadence, content, format, and scope of audit and feedback reports and care management team training). The implementation strategy intervention will then be refined by identifying the most acceptable approach to providing this implementation support to health centers. If results indicate the need to consider implementation strategies other than those proposed, the team will consider using them if feasible. The final set of implementation strategies will comprise the intervention to be tested in the study’s trial phase.

### Main Trial Study Design

**Figure 2.**
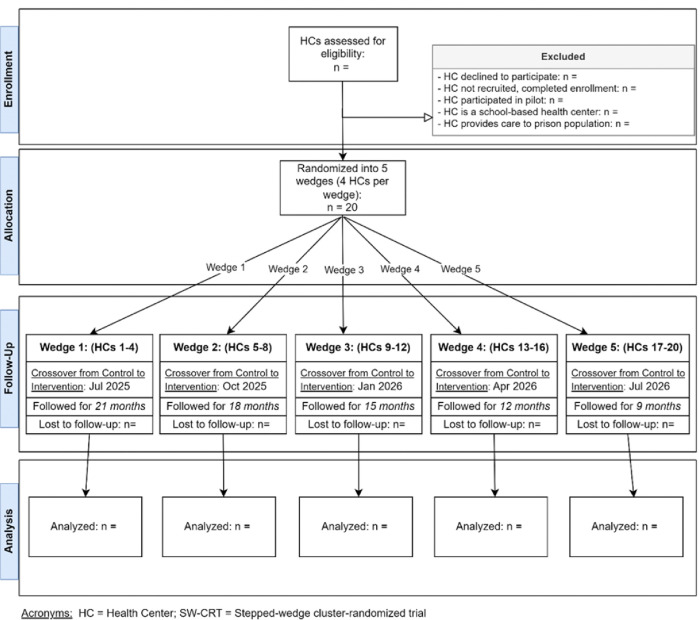
CONSORT SW-CRT Flowchart Diagram

Following the formative intervention development described above, a stepped-wedge cluster randomized trial (SW-CRT) will be conducted across 20 OCHIN member health centers, with health centers serving as the clusters. A CONSORT flow diagram outlining the randomization and intervention allocation is provided in Fig. 2.(65) In this trial, the CM teams within each health center will undergo a 12-week implementation support intervention. The stepped-wedge design enables the phased delivery of the intervention to small groups of health centers (four per wedge; five wedges). This approach optimizes study resources while maintaining methodological rigor, leveraging the higher statistical power in this design compared to other CRT designs.(50, 66–68) It also ensures that all participating health centers eventually receive the intervention, potentially enhancing study recruitment.

The 20 recruited health centers will be randomly assigned to one of five wedges using a random number generator independently by the study’s statistician with SAS software for staggered receipt of the intervention. The statistician will have no direct interaction with the health centers and will not be involved in the recruitment of these health centers into the study. Recruitment efforts will be led by the study’s Project Manager and Project Director. These health centers will initially start in the control condition, with each wedge sequentially transitioning (per randomization) to the intervention condition every three months. This process continues until all health centers receive the full 12-week intervention (Table 3). Before receiving the intervention, all study health centers will contribute at least six months of baseline data. After receiving the intervention, all study health centers will continue to be followed until the end of the main trial period, which will span from approximately January 2025 (start of baseline data collection) to June 2027 (end of follow-up for all wedges). The intervention will be delivered to health centers from July 2025 to September 2026. The final wedge to receive the implementation support will be followed for six months in the intervention condition. Participating health centers will receive an impact payment for their involvement.

### Outcomes

This hybrid implementation-effectiveness type 2 pragmatic trial has a mixed methods design. The primary outcomes include: 1) whether patients enrolled in care management programs have been screened for unmet social needs (binary); and 2) whether patients with unmet social needs received a referral to a community organization to address an identified social need (binary). The secondary outcomes include 1) receipt of community services (closed-loop referral) among patients with unmet social needs who were referred to community organizations (binary); and 2) clinical outcomes such as controlled hypertension (among patients with diagnosed hypertension) and controlled diabetes (among patients with diagnosed diabetes). Controlled hypertension is defined as having a blood pressure < 140/90 mmHg at their most recent visit. Controlled diabetes is defined as having hemoglobin A1c ≤ 9.0 percent at their most recent visit. Patients with hypertension and/or diabetes who had a visit but no hemoglobin A1c measurement or blood pressure measurement will be considered to have “uncontrolled” disease.(69) Table 4 outlines how each of the outcomes aligns with the RE-AIM framework.

### Inclusion criteria

Trial health centers will be recruited from OCHIN members that use CR for at least one care management or population health program that stipulates care coordination to address unmet social needs for a monthly average of ≥ 15 enrolled patients. Trial centers must also have care management staff to support programming and provide primary care.

### Data

All quantitative data will be extracted from the OCHIN EHR, including clinical-community care coordination activities performed by care management / population health providers using CR, and patients’ demographic, medical, laboratory, and social risk data.(70, 71) Qualitative data will come from periodic reflections and interviews with CM staff. Using similar methods as applied in formative activities, periodic reflections will be conducted at intervention period weeks 4 and 8. Semi-structured interviews with one key informant (e.g. care manager, health center champion) per health center will be conducted three to six months post intervention.

### Analyses

Primary outcomes will be measured at six time points for each health center: at baseline and at the end of each of the five wedge’s receipt of the intervention during the CRT. At each time point, all active CM patients receiving care management in a CR program that incorporates social risk activities (e.g. CalAIM, CCM) will be included. The analyses use an open cohort design, as some patients will be measured across multiple time points and others only once. We will identify whether each eligible patient has been screened. We will also identify if patients who screened positive for a social need were referred to a community organization; to the extent possible we will also identify which patients with social needs declined offered referrals. Currently, there is large variability in whether health centers collect data on patients who decline referrals. We will guide on collecting this information as part of implementation support, and we will investigate if we can assess declined referrals as part of our analyses. As the main outcomes are binary and patients are nested in health centers, we will use generalized linear mixed models with a binomial distribution and a logit link and include a random effect for the health center. Patient-level covariates will include age, race/ethnicity, gender, federal poverty level, insurance type, care management program (e.g. CCM; CalAIM), and a health center-level indicator of treatment arm (intervention or control) for each time point.

The analysis for closed-loop referral (secondary outcome) will be limited to patients enrolled in CM that received a referral to a community organization for a social need. Clinical outcomes of hypertension and diabetes control will be limited to enrolled patients with hypertension and/or diabetes, respectively, and a medical visit during the measurement period.

Qualitative data collected during and after the trial will be uploaded to NVivo QRS for data management and analysis. Analysis will be conducted following an immersion-crystallization approach, which entails multiple iterations of data immersion and analytic reflection to identify patterns in the data. First, the qualitative team will meet weekly to read periodic reflections and interview transcripts to develop inductive codes. As the identification of new codes slows and the team reaches agreement on code definition and appropriate application, the team will begin coding outside of weekly meetings. Continuing to meet to discuss potential revisions to codes and their definitions, the team prepares for in-depth analysis of queried codes. Once coding is complete, code queries will be generated for further analysis. In this step, reports from the queried codes are divided among team members for immersive analysis. The team will meet weekly to share content from analytic memos, outliers in the data, and begin to identify prospective themes. During this process, the qualitative team will bring emerging themes to the larger study team for further discussion, refinement, and sense-making. This process, which further strengthens analytic validity and rigor, will occur until interpretation saturation is reached.(72, 73) Informed by the 2019 National Academies of Sciences, Engineering, and Medicine report on integrating social and medical care,(8) our analysis will focus on understanding how individual, health center, and environmental factors interact to influence care management team use of CR to screen and refer for social needs and coordinate clinical-community linkages, particularly among groups that have been economically and socially marginalized. We will highlight factors related to intervention outcomes (e.g., reach, adoption, effectiveness) and generalizability of the implementation strategies and associated adjustments across health center contexts.

### Sample Size Calculation

We assessed power (66, 74–76) to detect differences in the percentage of patients with social needs who are referred to community organizations, which is a primary outcome of this study. We used a two-sided significance test with *α* = 0.05. The study’s pre-specified total sample size is 20 health centers with a stepped-wedge open cohort design(77) with five wedges each of four health centers (clusters). Based on preliminary data of high-acuity patients (defined as three or more chronic diseases on the Multimorbidity Weighted index(78–80)) and a completed social risk screen, the expected number of eligible patients is 163 per health center. This number represents the denominator for the primary outcome and the number of patients in each cluster. The proportion of patients with social needs who are referred to services (i.e., the numerator) varies widely, ranging from 10%(81) to 86% in the literature.(24) Therefore, we assume a baseline referral rate of 48%, which is the midpoint between the ranges. We also assume that the intervention would improve clinical-community linkages to between 63.3%(82) and 72%(23) based on other studies. To determine the power of this trial, we examined the absolute differences in screening rates for a range of intraclass correlations, which quantifies the degree of similarity in outcomes within each cluster (health center). We conservatively assumed a cluster autocorrelation and the individual autocorrelation of 0, as there are no sufficient data-driven estimates of these measures,(83) and a block exchangeable decay structure. The cluster autocorrelation refers to the correlation between the population means from the same group at two different periods, while the individual autocorrelation is the correlation between the outcome variable for the same individual at two different periods.(84) If the intervention improves screening rates to 63.3%, as observed in Lian et al. then we would have > 80% power to detect this difference with an intraclass correlation as high as 0.15. If the intervention improves screening rates to 72% as observed in Richman et. al, we would have > 80% to detect this difference with an intraclass correlation as high as 0.30. Power was calculated using the Shiny app(85) for the swCRTdesign(86) package in R.

## Discussion

Although social determinants are an important consideration for high-risk, medically complex care managed populations, evidence suggests that screening for social needs and providing linkages to community organizations to address identified patient unmet social needs can be hampered by barriers to enabling technologies. As health centers serving systematically underserved populations increase their capacity for documenting and addressing patients’ social needs, it is imperative to identify implementation strategies that support the adoption of enabling technologies and workflows to advance effective clinical-community linkages. While little is known about how to support implementation of technologies specifically focused on clinical-community linkages, evidence is available on implementation strategies that are effective in promoting adoption of other EHR-based tools. Refining these implementation strategies with health center input is expected to support improving the intervention’s ability to conduct social risk screening and place needed referrals among high-risk patients. This is time sensitive, as chronic disease guidelines and value-based payor models are increasingly stipulating screening, referring, and supporting receipt of services for patients’ social needs. To our knowledge, this is the first study to test a set of implementation strategies to improve clinical-community linkages among safety-net primary care health centers. Results will be disseminated to guide primary care clinics on using implementation strategies to further adoption of enabling technologies targeting social risk-targeted care via clinical-community linkages.

### Limitations and Considerations

This pragmatic trial will be conducted with a focus on one enabling technology, CR, an application in the Epic EHR. To our knowledge, CR is the first instance of a seamlessly integrated EHR module designed to meet the unique, complex needs of care coordinators providing care to health disparate patients within primary care settings. As social risk-targeted care is advanced through care guidelines and payors, it would be expected that additional applications will be developed in other EHR systems. To address this, disseminated study findings will be presented in an EHR-agnostic manner. Additionally, the study design does not directly address other known factors that influence clinical-community linkages including funding, staff resources, community organizational capacity, and patient preferences. Our team will work closely with health centers and community partners to identify the broadest possible range of internal and external factors that may influence outcomes and will consider these in results interpretation and dissemination.

## Conclusions

No prior studies have assessed how EHR-enabled clinical-community linkages can be effectively operationalized in safety-net primary care health centers. This will be the first study to develop a set of implementation strategies targeting the adoption of enabling technologies to promote social risk screening, referrals, and service receipt assessment. Results have the potential to improve clinical-community linkages and advance health equity for the over 30 million low-income and racial/ethnic minority populations cared for in community-based health centers.

### Trial Status

The study was reviewed by the Advarra Institutional Review Board (Pro00074727) which provided approval for quantitative data collection and analysis and the modified Delphi, semi-structured interviews, and guided tour of EHR that comprise formative activities. ). (Aim 1 and 2: January 15, 2024; Aim 3: October 20, 2023). The institutional review board will receive the protocol again before the pilot and main trial. No research with human subjects will be conducted until the proper approvals have been received.

## Figures and Tables

**Table 1:** Study concepts and approach mapped to ITIM concepts

ITIM Concepts	Applied in study	Description
	
**INNER CONTEXT**		

Technology adoption (Dependent)	Primary Outcome	Social needs screening and referring among patients in CM programs

Implementation	Intervention	Refinement and testing of implementation strategies will address technology-driven facilitators and barriers to align clinic environment, needs, and policies with clinical-community linkages

Technology	EHR application targeted by intervention	Enabling technology (CR) has been adopted to facilitate linkages to community organizations. The intervention will optimize technology and workflows to support clinical-community linkages

Interfacing systems	EHR application targeted by intervention	Clinical-community linkages should be user-friendly and seamless

Workflow old	Analysis	Qualitative analysis will assess old workflows and identify areas for optimization of clinical-community linkage workflows

Workflow new	Intervention	Practice facilitation is a key implementation strategy of the intervention

Users (adopters)	Analysis	Understanding user needs through health center engagement will inform refinement of intervention

Leadership	Intervention	Championship is a key implementation strategy of the intervention

Communication	Intervention	Audit & Feedback and implementation support by Practice Coach, Champion, and Trainers are key implementation strategies of the intervention

**OUTER CONTEXT**		

Accreditation/regulation	EHR application targeted by intervention	Reimbursement of social needs screening, referral and community organization linkage are important components of CM and emerging value-based care programs

Economic environment	Outcome	Clinics will need to demonstrate their ability to support clinical-community linkages to be competitive for emerging value-based care contracts

Facilitators	Intervention	Broad health center partner engagement will inform implementation strategies that will comprise the intervention to improve clinical-community linkages

Vendor	EHR application/tools targeted by intervention	Epic is an EHR vendor and tool developer that supports the operationalization of clinical-community linkages

**Table 2:** Role of formative activities on refining implementation strategies

Implementation strategy	*Formative activities will provide needed input in the following areas to refine the intervention for live testing in health centers*:		
	Content	Format	Cadence
**Dedicated training** to foster adoption and efficient use of enabling technologies to support social needs care coordination activities(34)	- Topics to cover at initial training - Ongoing training content	- Individual or group - Synchronous, asynchronous or hybrid	- Length of initial training - Frequency of booster trainings
**Clinic champions** promote buy-in and engagement by building internal support and mobilizing resources(87)	- Recruitment criteria for champion - Scope of champion role	- On-site assessment of adoption by users - Communication preferences with study team	- Hours per week needed to accomplish champion activities
**Practice coaches** facilitate workflow redesign to optimize adoption of enabling technologies(88)	- Focus within one department or across departments - Coordinate with ongoing internal and external initiatives to screen, refer, and ascertain receipt of services	- Individual or group - Synchronous, asynchronous or hybrid	- Hours of initial practice coaching support needed by health center - Frequency of meetings through implementation
**Audit & feedback** reports show progress and evidence needed to facilitate benchmarking and motivate users(89)	- Determine data to display to show progress and adoption of enabling technologies and receipt of community services (closed-loop referrals)	- Use of graphs, tables, and text to convey information	- Frequency of report creation, distribution, and review

**Table 3:** Stepped-wedge design

					Year 1	Year 2		Year 3				Year 4				Year 5			
Q1	Q2	Q3	Q4	Q1	Q2	Q3	Q4	Q1	Q2	Q3	Q4	Q1	Q2	Q3	Q4	Q1	Q2	Q3	Q4
Wedge 1 (Health Centers 1–4)					Formative Intervention Refinement	C	C	I	F	F	F	F	F	F	Analysis and Dissemination				
Wedge 2 (Health Centers 5–8)						C	C	C	I	F	F	F	F	F					
Wedge 3 (Health Centers 9–12)						C	C	C	C	I	F	F	F	F					
Wedge 4 (Health Centers 13–16)						C	C	C	C	C	I	F	F	F					
Wedge 5 (Health Centers 17–20)						C	C	C	C	C	C	I	F	F					

Note: C = Control condition, I = Intervention condition, F = Follow-up.

**Table 4:** Outcomes Per RE-AIM Framework.

RE-AIM Outcome	Outcome Type	Measure
Reach	Primary	binary outcome of whether the patient has been screened for unmet social needs
Effectiveness	Primary Secondary Secondary	binary outcome of whether the patient with an unmet social need received a referral to a community organization to address an identified social need binary outcome of controlled hypertension binary outcome of controlled diabetes
Adoption	Secondary	binary outcome of receipt of community services (closed-loop referral) among patients with a social need who were referred to community organizations
Implementation	Secondary	*assessed through qualitative methods
Maintenance	Secondary	*assessed through qualitative methods

## Data Availability

The data sets generated and analyzed during this study are not publicly available due to sourcing patient-level data from multiple health systems, which have restrictions regarding the availability and rerelease of data under cross-institution agreements. Data are available from the corresponding author on reasonable request and with permission of all relevant parties.

## Abbreviations

CalAIM ECM: California Advancing and Innovating Medi-Cal Enhanced Care Management
CCM: Chronic Care Management
CM: Care management
CMS: Centers for Medicare and Medicaid
CRT: Cluster randomized trial
EHR: Electronic health record
HIT: Health information technologies
ITIM: Integrated Technology Implementation Model
NIH: National Institutes of Health
PCM: Principal Care Management
PCMH: Patient-Centered Medical Home
RE-AIM QuEST: Reach, Effectiveness, Adoption, Implementation and Maintenance Qualitative Evaluation for Systematic Translation
SDH: Social determinants of health
SW-CRT: Stepped wedge cluster randomized trial
TCM: Transitional Care Management
